# Supplementary material for: Multi-omics analysis of pyroptosis regulation patterns and characterization of tumor microenvironment in patients with hepatocellular carcinoma
Source: PeerJ. 2023 May 11;11:e15340. doi: 10.7717/peerj.15340 (PMC10183172; doi:10.7717/peerj.15340)
Supplement: Supplemental Information 1 [file peerj-11-15340-s001.docx]

**Table S1: TCGA-HCC patient baseline table.**

|  | **high risk** | **low risk** | **p** | **test** |
| --- | --- | --- | --- | --- |
| **n** | **163** | **163** |  |  |
| **group = low risk (%)** | **0 (0.0)** | **163 (100.0)** | **<0.001** | **exact** |
| **age = 60+ (%)** | **83 (50.9)** | **90 (55.2)** | **0.506** | **exact** |
| **stage_M = MX (%)** | **36 (22.1)** | **39 (23.9)** | **0.793** | **exact** |
| **stage_N (%)** |  | **0.234** | **exact** |  |
| **N0** | **120 (73.6)** | **113 (69.3)** |  |  |
| **N1** | **0 (0.0)** | **3 (1.8)** |  |  |
| **NX** | **43 (26.4)** | **47 (28.8)** |  |  |
| **stage_T (%)** |  | **0.009** | **exact** |  |
| **T1** | **99 (60.7)** | **71 (43.6)** |  |  |
| **T2** | **34 (20.9)** | **45 (27.6)** |  |  |
| **T3** | **27 (16.6)** | **40 (24.5)** |  |  |
| **T4** | **2 (1.2)** | **7 (4.3)** |  |  |
| **TX** | **1 (0.6)** | **0 (0.0)** |  |  |
| **gender = male (%)** | **117 (71.8)** | **107 (65.6)** | **0.282** | **exact** |
| **race (%)** |  |  | **0.262** | **exact** |
| **american indian or alaska native** | **1 (0.6)** | **0 (0.0)** |  |  |
| **asian** | **75 (46.0)** | **75 (46.0)** |  |  |
| **black or african american** | **7 (4.3)** | **6 (3.7)** |  |  |
| **not** reported | **8 (4.9)** | **2 (1.2)** |  |  |
| **white** | **72** (44.2) | **80** (49.1) |  |  |
| **tumor_stage (**%) |  | **0.008** | **exact** |  |
| **not reported** | **7 (4.3)** | **10 (6.1)** |  |  |
| **stage i** | **95 (58.3)** | **65 (39.9)** |  |  |
| **stage ii** | **33 (20.2)** | **41 (25.2)** |  |  |
