# Supplementary material for: Multi-omics analysis of pyroptosis regulation patterns and characterization of tumor microenvironment in patients with hepatocellular carcinoma
Source: PeerJ. 2023 May 11;11:e15340. doi: 10.7717/peerj.15340 (PMC10183172; doi:10.7717/peerj.15340)
Supplement: Supplemental Information 2 [file peerj-11-15340-s002.docx]

**Table S2: Go enrichment analysis of differentially expressed genes between high-risk and low-risk groups.**

| **Category** | **ID** | **Description** | **pvalue** |
| --- | --- | --- | --- |
| BP | GO:0007586 | digestion | 2.52E-09 |
| BP | GO:0030277 | maintenance of gastrointestinal epithelium | 2.83E-06 |
| BP | GO:0022600 | digestive system process | 1E-05 |
| BP | GO:0010669 | epithelial structure maintenance | 1.2E-05 |
| BP | GO:0016266 | O-glycan processing | 4.38E-05 |
| BP | GO:0098742 | cell-cell adhesion via plasma-membrane adhesion molecules | 0.000159 |
| BP | GO:0042471 | ear morphogenesis | 0.000167 |
| BP | GO:0046879 | hormone secretion | 0.000378 |
| BP | GO:0048568 | embryonic organ development | 0.000451 |
| BP | GO:0001894 | tissue homeostasis | 0.000455 |
| MF | GO:0099095 | ligand-gated anion channel activity | 0.000898 |
| MF | GO:0005254 | chloride channel activity | 0.00123 |
| MF | GO:0005179 | hormone activity | 0.002106 |
| MF | GO:0005253 | anion channel activity | 0.002396 |
| MF | GO:0048018 | receptor ligand activity | 0.002464 |
| MF | GO:0030246 | carbohydrate binding | 0.002533 |
| MF | GO:0030546 | signaling receptor activator activity | 0.002738 |
| MF | GO:0022836 | gated channel activity | 0.003724 |
| MF | GO:0015276 | ligand-gated ion channel activity | 0.004027 |
| CC | GO:0016342 | catenin complex | 0.000397 |
| CC | GO:0098982 | GABA-ergic synapse | 0.000681 |
| CC | GO:0005796 | Golgi lumen | 0.000909 |
| CC | GO:0032590 | dendrite membrane | 0.00124 |
| CC | GO:0045177 | apical part of cell | 0.002132 |
| CC | GO:0034707 | chloride channel complex | 0.00218 |
| CC | GO:0099056 | integral component of presynaptic membrane | 0.003304 |
| CC | GO:0032589 | neuron projection membrane | 0.003991 |
| CC | GO:0099055 | integral component of postsynaptic membrane | 0.004901 |
