# Supplementary material for: Multi-omics analysis of pyroptosis regulation patterns and characterization of tumor microenvironment in patients with hepatocellular carcinoma
Source: PeerJ. 2023 May 11;11:e15340. doi: 10.7717/peerj.15340 (PMC10183172; doi:10.7717/peerj.15340)
Supplement: Supplemental Information 3 [file peerj-11-15340-s003.docx]

**Table S3: KEGG enrichment analysis of DEGs of differentially expressed genes between high-risk and low-risk groups.**

| **Category** | **ID** | **Description** | **pvalue** |
| --- | --- | --- | --- |
| KEGG_PATHWAY | hsa04080 | Neuroactive ligand-receptor interaction | 5.63E-05 |
| KEGG_PATHWAY | hsa04742 | Taste transduction | 0.001914 |
| KEGG_PATHWAY | hsa04972 | Pancreatic secretion | 0.004025 |
| KEGG_PATHWAY | hsa00430 | Taurine and hypotaurine metabolism | 0.005494 |
| KEGG_PATHWAY | hsa04024 | cAMP signaling pathway | 0.007434 |
| KEGG_PATHWAY | hsa04540 | Gap junction | 0.012952 |
| KEGG_PATHWAY | hsa00600 | Sphingolipid metabolism | 0.014045 |
| KEGG_PATHWAY | hsa04657 | IL-17 signaling pathway | 0.016168 |
| KEGG_PATHWAY | hsa04514 | Cell adhesion molecules | 0.01897 |
| KEGG_PATHWAY | hsa04530 | Tight junction | 0.030624 |
