# Supplementary material for: Multi-omics analysis of pyroptosis regulation patterns and characterization of tumor microenvironment in patients with hepatocellular carcinoma
Source: PeerJ. 2023 May 11;11:e15340. doi: 10.7717/peerj.15340 (PMC10183172; doi:10.7717/peerj.15340)
Supplement: Supplemental Information 4 [file peerj-11-15340-s004.docx]

**Table S4: GSEA analysis between high-risk and low-risk groups (inhibited biological function).**

| ID | ES | NES | pvalue |
| --- | --- | --- | --- |
| go_inhibitory_extracellular_ligand_gated_ion_channel_activity | -0.85224 | -1.93587 | 9.71E-05 |
| go_limb_bud_formation | -0.83795 | -1.9034 | 0.000239 |
| go_condensed_chromosome_outer_kinetochore | -0.83151 | -1.97688 | 5.58E-05 |
| go_spindle_elongation | -0.82814 | -1.83236 | 0.000416 |
| go_regulation_of_chloride_transport | -0.80699 | -1.78556 | 0.001196 |
| go_mitotic_dna_replication | -0.79769 | -1.9836 | 9.96E-05 |
| go_synaptic_vesicle_docking | -0.79619 | -1.80854 | 0.001997 |
| go_chromatoid_body | -0.78929 | -1.8765 | 0.000609 |
| go_negative_regulation_of_transcription_by_competitive_promoter_binding | -0.78684 | -1.74097 | 0.002793 |
| go_galactolipid_metabolic_process | -0.77716 | -1.71956 | 0.003771 |
| kegg_cell_cycle | -0.50829 | -1.75203 | 3.25E-05 |
| kegg_ecm_receptor_interaction | -0.48663 | -1.61201 | 0.004043 |
| kegg_neuroactive_ligand_receptor_interaction | -0.46544 | -1.67554 | 2.41E-06 |
| kegg_gap_junction | -0.46196 | -1.53597 | 0.007855 |
| kegg_axon_guidance | -0.44686 | -1.54182 | 0.003792 |
| kegg_huntingtons_disease | 0.379055 | 1.8326 | 5.28E-06 |
| kegg_steroid_hormone_biosynthesis | 0.390165 | 1.552235 | 0.009584 |
| kegg_alzheimers_disease | 0.400588 | 1.910789 | 1.57E-06 |
| kegg_arachidonic_acid_metabolism | 0.402226 | 1.612863 | 0.008925 |
| kegg_ppar_signaling_pathway | 0.449864 | 1.845516 | 0.000117 |
