# Supplementary material for: Multi-omics analysis of pyroptosis regulation patterns and characterization of tumor microenvironment in patients with hepatocellular carcinoma
Source: PeerJ. 2023 May 11;11:e15340. doi: 10.7717/peerj.15340 (PMC10183172; doi:10.7717/peerj.15340)
Supplement: Supplemental Information 5 [file peerj-11-15340-s005.docx]

**Table S5: GSEA analysis between high-risk and low-risk groups (promoted biological functions).**

| ID | ES | NES | pvalue |
| --- | --- | --- | --- |
| go_very_low_density_lipoprotein_particle_remodeling | 0.887434 | 2.390189 | 1.98E-06 |
| go_triglyceride_rich_lipoprotein_particle_remodeling | 0.885009 | 2.464035 | 3.42E-07 |
| go_complement_activation_lectin_pathway | 0.873119 | 2.389054 | 2.67E-06 |
| go_platelet_dense_granule_lumen | 0.871294 | 2.42585 | 9.9E-07 |
| go_alcohol_dehydrogenase_nad_p_plus_activity | 0.86067 | 2.2559 | 2.65E-05 |
| go_chylomicron | 0.847338 | 2.359152 | 6.56E-06 |
| go_glyoxylate_metabolic_process | 0.844408 | 2.213275 | 6.14E-05 |
| go_blood_coagulation_intrinsic_pathway | 0.837189 | 2.530543 | 2E-07 |
| go_fatty_acid_beta_oxidation_using_acyl_coa_dehydrogenase | 0.83296 | 2.183269 | 0.000118 |
| go_urea_cycle | 0.83096 | 2.238084 | 0.000121 |
| kegg_primary_bile_acid_biosynthesis | 0.847215 | 2.524414 | 2.89E-07 |
| kegg_fatty_acid_metabolism | 0.765637 | 2.896549 | 1E-10 |
| kegg_complement_and_coagulation_cascades | 0.738595 | 3.04783 | 1E-10 |
| kegg_glycine_serine_and_threonine_metabolism | 0.711765 | 2.544408 | 2.27E-07 |
| kegg_renin_angiotensin_system | 0.669718 | 2.062439 | 0.001817 |
| kegg_valine_leucine_and_isoleucine_degradation | 0.667422 | 2.542512 | 8.42E-08 |
| kegg_peroxisome | 0.639025 | 2.697852 | 1E-10 |
| kegg_histidine_metabolism | 0.625501 | 2.165818 | 0.000229 |
| kegg_parkinsons_disease | 0.608925 | 2.673643 | 1E-10 |
| kegg_ribosome | 0.604297 | 2.580318 | 1E-10 |
