# Supplementary material for: Multi-omics analysis of pyroptosis regulation patterns and characterization of tumor microenvironment in patients with hepatocellular carcinoma
Source: PeerJ. 2023 May 11;11:e15340. doi: 10.7717/peerj.15340 (PMC10183172; doi:10.7717/peerj.15340)
Supplement: Supplemental Information 7 [file peerj-11-15340-s007.docx]

Test plan

Multi-omics Analysis of Pyroptosis Regulation Patterns and Characterization of Tumor Microenvironment in Patients with hepatocellular carcinoma.

Research process：

In this study, 80 patients with liver cancer diagnosed for the first time in our hospital (except those who received preoperative radiotherapy and chemotherapy and died of surgical complications) are planned to be enrolled, and the intraoperative liver tissues (liver cancer and normal liver tissues around the cancer) of patients with liver cancer from January to December 2021 are collected.

(1) The study plans to include 40 patients. During the operation, fresh liver tissues (liver cancer and normal liver tissues around the cancer) of liver patients were used to study the distribution of scorch related molecules mRNA in cancer and adjacent normal liver tissues by grinding tissue samples, extracting RNA, reverse transcription, and using qPCR method to detect the expression content of scorch related molecules mRNA in cancer tissues and normal liver tissues around the cancer.

① Total RNA extraction from tissues

② RNA reverse transcription

③Real-time PCR

④ Statistical analysis of qPCR results

(2) In this study, 40 patients were enrolled in the study. Postoperative paraffin embedded liver tissue samples (liver cancer and normal liver tissue around the cancer) of patients with liver cancer were stained with HE. Immunohistochemical methods were used to detect the expression of scorch related molecular proteins. To study the clinical significance of scorch related molecular protein in human hepatocellular carcinoma, and analyze the relationship between its expression and tumor microenvironment and prognosis. The specific test methods are as follows:

① Immunohistochemical staining

② Statistical analysis of immunohistochemical results

(2) In this study, 40 patients were enrolled in the study. Postoperative paraffin embedded liver tissue samples (liver cancer and normal liver tissue around the cancer) of patients with liver cancer were stained with HE. Immunohistochemical methods were used to detect the expression of scorch related molecular proteins. To study the clinical significance of scorch related molecular protein in human hepatocellular carcinoma, and analyze the relationship between its expression and tumor microenvironment and prognosis. The specific test methods are as follows:

① Immunohistochemical staining

② Statistical analysis of immunohistochemical results
